# Analysis of genotype by methylation interactions through sparsity-inducing regularized regression

**DOI:** 10.1186/s12919-018-0145-6

**Published:** 2018-09-17

**Authors:** Wenda Zhou, Shaw-Hwa Lo

**Affiliations:** Department of Statistics, Columbia University, 1255 Amsterdam Avenue, New York, NY 10027 USA

## Abstract

In this paper, we consider the use of the least absolute shrinkage and selection operator (LASSO)-type regression techniques to detect important genetic or epigenetic loci in genome-wide association studies (GWAS) and epigenome-wide association studies (EWAS). We demonstrate how these techniques can be adapted to provide quantifiable uncertainty using stability selection, including explicit control of the family-wise error rate. We also consider variants of the LASSO, such as the group LASSO, to study genetic and epigenetic interactions. We use these techniques to reproduce some existing results on the Genetics of Lipid Lowering Drugs and Diet Network (GOLDN) data set, which collects from 991 individuals blood triglyceride and differential methylation at 464,000 cytosine-phosphate-guanine (CpG) sites and 761,000 single-nucleotide polymorphisms (SNPs), and to identify new research directions. Epigenome-wide and genome-wide models based on the LASSO are considered, as well as an interaction model limited to chromosome 11. The analyses replicate findings concerning 2 CpGs in carnitine palmitoyltransferase 1A (CPT1A). Some suggestions are made regarding potentially interesting directions for the analysis of genetic and epigenetic interactions.

## Background

Blood lipids are linked to increased risk of heart disease, heart attack and stroke. Researchers have explored the effects of genetic variation [1] and epigenetic variation [2] on lipid metabolism. Both these studies use traditional F-statistics to screen for important loci of genetic or epigenetic variation. Such statistics, however, tend to lose power when faced with the high-dimensional nature of these problems, as adjusting for multiple comparisons is necessary.

We propose an alternative variable screening method that considers a more global approach. Adaptations of the least absolute shrinkage and selection operator (LASSO) selector, first proposed by Tibshirani [3], have proven powerful in the context of high-dimensional variable selection. We combine such a method with the strategy of stability selection [4] to obtain a global selection strategy that enables us to control the family-wise error rate. We apply these methods to study both genetic and epigenetic effects on blood lipids. Finally, we attempt to extend such methods to tackle the genetic–epigenetic interactions and their effects on blood lipids.

## Methods

### LASSO and sparse regression

A popular tool to tackle statistical problems with a large number of covariates is the LASSO. First proposed by Tibshirani [3], its computational tractability and ability to produce sparse responses has made it very popular in the biomedical field, and in problems such as genome-wide association studies (GWAS) and similar.

The LASSO and its variants attempt to solve a sparse linear regression problem by optimizing a penalized least-squares objective function. Given a vector *y* of responses, and a design matrix *X*, the LASSO estimator is the solution of $\underset{\beta }{\min}\frac{1}{2n}{\left\Vert y- X\beta \right\Vert}^2+\lambda \Omega \left(\beta \right),$where *Ω*(*β*) is a sparsity-inducing prior; for example, Ω(*β*) = ‖*β*‖_1_ = ∑_*i*_ ∣ *β*_*i*_∣ in the case of the LASSO. Here, *λ* > 0 is a tuning parameter that trades off between goodness-of-fit and sparsity of the solution.

We note that in practice, it may be helpful to add a slight *l*_2_ penalty in addition to the sparsity-inducing penalty to reduce the effect of correlation among predictors. Such a procedure, called *elastic-net*, was first introduced by Zou and Hastie [5], and often displays superior performance in simulations [6, 7]. The LASSO and its variations have been shown to obtain good theoretical properties [8, 9], as well as good performance on GWAS data sets [6].

### Regression model and family structure

#### Kinship

It is common to model the outcome as a simple univariate linear regression where the outcome is regressed on a particular single-nucleotide polymorphism (SNP) or cytosine-phosphate-guanine (CpG) location, and covariates are included in the regression to control for environmental effects such as smoking. In such a context, it is often important to model the dependency among the remaining unobserved genetic information of individuals who are related, that is, the kinship.

This paper follows a different approach by jointly modeling the effect of all the SNP and/or CpG locations in the regression. The dependency between related individuals resulting from unaccounted genetic or epigenetic correlation is thus greatly reduced. In particular, the need to model kinship is greatly reduced, and it was not modeled in this paper.

We note that this does not control for any correlation resulting from environmental effects, such as individuals who live in the same household. We were unable to take such effects into account as household information was unavailable.

### Linear allele effect

A common assumption in GWAS studies is that of a *linear allele effect*, (LAE) that is, the effect of the presence of 2 minor alleles is twice that of the presence of single minor allele: the SNP is included in the linear regression as a single numerical variable corresponding to the number of minor alleles. In this paper, interactions between the methylation at CpG sites and SNPs are of particular interest, which makes this assumption more tenuous than it usually is. Indeed, in the linear regression framework it is natural to model the interaction as a multiplicative interaction, which would further imply that not only is the presence of the minor allele linear, so is the interaction between methylation and allele.

Instead, we consider a fully general model where the allele value is coded in a categorical fashion using indicator variables. Although this may cause a loss of power by increasing the number of variables, penalized regression techniques such as LASSO and group LASSO are somewhat able to mitigate this.

### Stability selection

In addition to obtaining a binary answer (whether a locus was selected), it is often of interest to quantify the uncertainty in the selection. Although significance tests have been developed for the LASSO [10], their interpretation is delicate given the problem of selecting the tuning parameter *λ*.

We chose a different method to quantify the uncertainty that is inspired by the bootstrap and similar resampling methods: stability selection. Stability selection was first proposed by Meinshausen and Bühlmann [4], and subsequently improved by Shah and Samworth [11]. It is a technique that may be used to adapt any variable selection technique to produce a statistical testing procedure for which the Type I error may be characterized; for example, by controlling the per-family error rate [12].

Stability selection attempts to select variables (or groups of variables) that are “robust” to perturbations in the data set. To apply stability selection to a given variable selection method, each method was run numerous times on modified data sets containing a random subsample (without replacement) of the observations and with the penalty randomly rescaled for each variable. More precisely, as suggested in Shah and Samworth [11], random subsamples were taken to be of a size that was exactly half of our total data set, and whenever a subsample was included, so was its “complementary pair”; that is, those observations that were left out.

One of the main advantages of stability selection is that it enables us to control the family-wise error rate with only very weak assumptions on our selection procedure, reducing our dependence on selecting the appropriate penalization and other tuning parameters for the LASSO. Indeed, if variables are deemed significant whenever their probability of selection under the stability selection procedure is greater than a given threshold *τ*, the number of falsely selected variables *V* can be controlled in expectation (under technical assumptions) by $\mathbb{E}V\le pM\left(\tau, q,p\right),$ where *p* is the total number of variables, *q* is the number of variables selected on average in each random sample, and *M* is some (known and computable) function. For the rigorous definition and statement, see Shah and Samworth [11]. Hofner et al. [13] provides extensive simulation results.

### Group LASSO for categorical variables and interactions

The group LASSO [12] modifies the LASSO penalty *Ω* to penalize groups of variables together. Suppose that we have *n*_*g*_ groups, and let *β*_*j*_, *j* = 1, …, *n*_*g*_ be the coefficients for each group (${\beta}_j\in {\mathbb{R}}^{d_j}$ where group *j* has *d*_*j*_ variables), then the group LASSO is the solution of the optimization problem:

$$ \underset{\beta }{\min}\frac{1}{2n}{\left\Vert y- X\beta \right\Vert}_2^2+\lambda {\sum}_j{\left\Vert {\beta}_j\right\Vert}_2 $$

We note that although we are using the two-norm penalty ‖*β*_*j*_‖_2_, it does not appear squared, and hence does indeed induce sparsity at a group level: it is the case that either each element of *β*_*j*_ = 0, or that each element of *β*_*j*_ ≠ 0. Note that this generalizes the standard LASSO, which corresponds to the group LASSO with groups of size 1.

This adaptation of the LASSO allows us to include categorical variables into the regression model. Indeed, to include a categorical variable *X*_*j*_ as a linear regressor, it is necessary to use some form of indicator coding, which causes a categorical regressor to expand into several indicator columns corresponding to levels of the categorical variable. Group LASSO enables us to accommodate such practice by including these indicator variables as a group and selecting either all or none of them.

Even though the LASSO is particularly adapted for high-dimensional problems, finding interactions in such data sets is challenging. Some proposals (eg, Hofner et al. [13]) adopt a 2-step process by screening for variables with marginal importance first. However, it occasionally can be the case that variables of little marginal importance may be highly significant once interactions are included, making it desirable to consider such interactions together. Yuan and Lin [14] propose leveraging the group LASSO to screen for interactions that respect the strong hierarchy. Indeed, given 2 variables—*X*_1_, *X*_2_—for which we would like to include an interaction term, we may include a group consisting of the variables [*X*_1_, *X*_2_, *X*_1_ ∗ *X*_2_]. Note that some variables may appear in several groups, which is often called *overlapped group LASSO* [15–17]. We can recover the effect of such variables by simply summing all of their coefficients.

In addition, we note that in the case of methylation by genotype interactions, there is a natural biological structure to the problem. By considering the location of the methylation and genetic mutation sites on the chromosome, we may restrict the space of our interactions by requiring them to be colocated on the chromosome.

## Results and discussion

### Epigenetic association

We first consider the analysis of the effect of methylation on blood lipids as in Irvin et al. [2]. We regress the log of the mean fasting triglyceride (TG) level on the logit of the methylation, while controlling for the age and smoking status of each patient and the scores of the first four principal components of the methylation. The number of principal components is chosen as in Irvin et al. [2] so that the results are comparable, and the regression is done epigenome-wide.

We use an elastic-net and select the penalization by cross-validation on the data set. We subsequently apply the conjugate pair stability selection to 100 random pairs, and obtain the observed selection probability. The procedure is done using a custom-written group LASSO solver implementing block-coordinate descent [18] in Python. With the cross-validated penalty, there are an average of 112 selected CpG sites for a given stability-selection sample.

**Fig. 1:**
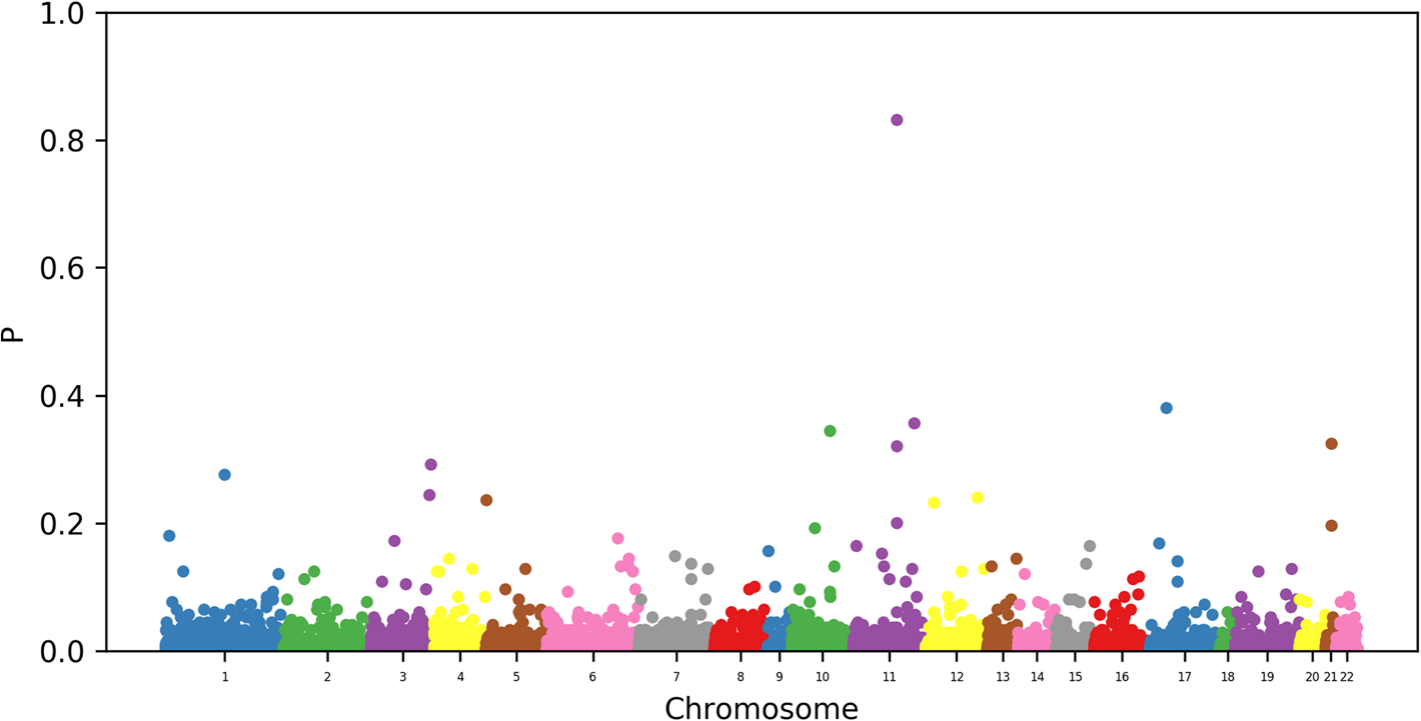
Probability that a CpG site is selected in the epigenome-wide model

We have reproduced in Fig. 1 a similar Manhattan plot as in Irvin et al. [2] by plotting the log probability that a given locus is not selected. Note that although these numbers may be viewed as a measure of confidence, they are not *p* values and cannot be interpreted as such.

We observe a result similar to that in Irvin et al. [2]. In particular, we observe that 2 of the CpG sites reported as significant by Irvin et al. [2] related to the carnitine palmitoyltransferase 1A gene *CPT1A* appear as significant in our data, as shown in Table 1. In this case, the cutoff to control the per-family error rate at a level of 0.05 is given by *τ* = 0.33. We also note that 2 of the CpG sites are contained within genes (*SREBF1* and *APAOA5*) that are related to the regulation of lipid metabolism by the peroxisome proliferator activated receptor α (PPARα) pathway.

**Table 1:** The most frequently selected CpG marks

Mark name	Gene	Selection probability
cg00574958	*CPT1A*	0.92
cg11024682	*SREBF1*	0.54
cg12556569	*APOA5*	0.51
cg07504977	N/A	0.48
cg06500161	*ABCG1*	0.47
cg27452255	N/A	0.44
cg26797124	N/A	0.43
cg17058475	*CPT1A*	0.42
cg24819835	*CD38*	0.42
cg17287155	*AHRR*	0.40

### Genetic association

**Fig. 2:**
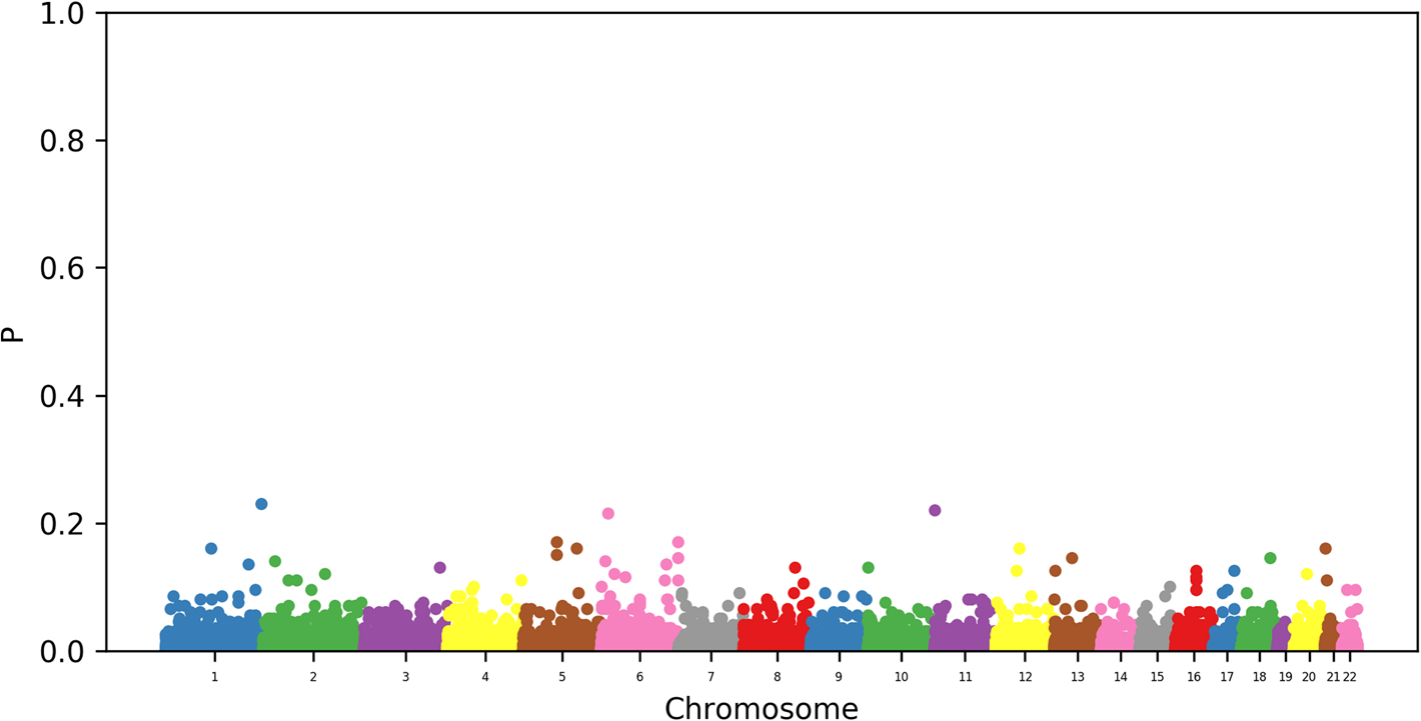
Probability that a SNP is selected in the genome-wide model

We now consider the analysis of the association of blood lipids and genotype. The log of the mean fasting TG level is regressed on a feature matrix created from the genotype, controlling for age, smoking, and the scores of the first four principal components of the genotype. The regression is done using the group LASSO with a slight elastic-net penalization selected by cross-validation. Each SNP is coded using indicators that are considered as a group for the purpose of the group LASSO. Conjugate pair stability selection was applied on 100 random pairs, and the observed selection probability of the groups were obtained. The cross-validated penalty yielded an average of 230 selected groups. We produce a similar Manhattan plot as previously (Fig. 2).

We note that the signal we obtain is significantly weaker than for the epigenetic model. However, we obtain a similar ranking for both the linear allele effect model and the fully general model, as can be seen in Table 2. We note that the *FAM120B* gene is related to the PPARα pathway.

**Table 2:** The most frequently selected SNPs

SNP	Gene	LAE selection probability (rank)	General selection probability (rank)
rs7765549	*NEDD9*	0.31 (1)	0.17 (3)
rs10514174	*THBS4*	0.30 (2)	0.17 (4)
rs16901314	*FAM120B*	0.28 (3)	0.16 (5)
rs2064039	N/A	0.27 (4)	0.16 (7)
rs17769833	N/A	0.26 (6)	0.15 (9)
rs17027070	*DENND2D*	0.25 (8)	0.16 (6)

### Interactions

Finally, effects of epigenetic–genetic interactions on mean fasting TG levels were considered. Because of the high dimension of the search space, only chromosome 11 is considered, where the most significant epigenetic effects were observed. To limit the number of possible interactions, only interactions between methylation and SNP loci that are separated by less than 0.01% of the chromosome were considered (approximately 10,000 base pairs). Although this is adequate to model local variations in the methylation mediating gene expression, one may also be interested in taking further steps toward exploring interactions among genetic pathways that are not local to a single chromosome.

The constructed feature matrix has 28,285 groups of size 1, corresponding to the methylation loci, 36,796 groups of size 2, corresponding to the SNP loci, and 190,073 groups of size 5, corresponding to the interactions of nearby methylation and SNP loci.

The log of the mean fasting TG level is regressed on the feature matrix described above using the group LASSO with the described structure additionally controlling for age, smoking, the scores of the first four principal components of the methylation, and the scores of the first four principal components of the genotype.

**Fig. 3:**
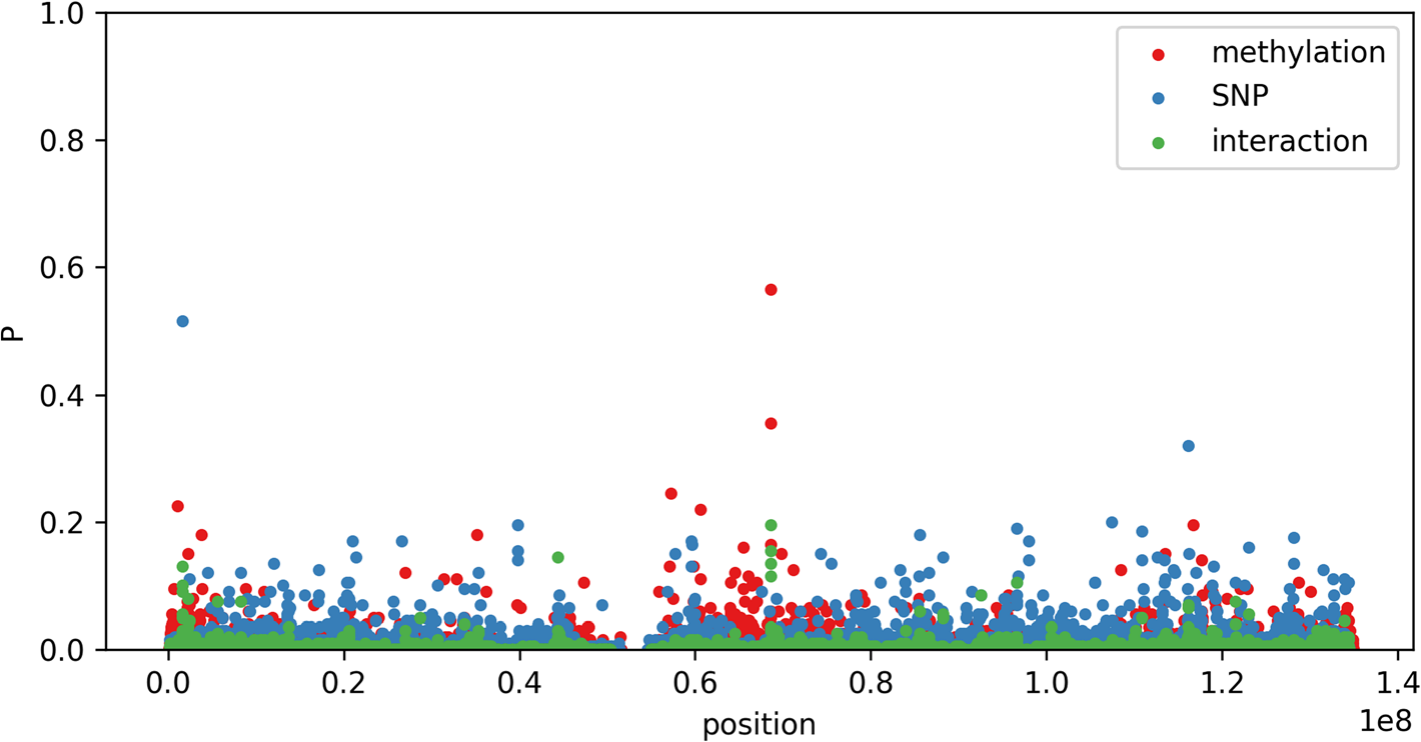
Probability that a CpG site, SNP, or interaction is selected in the interaction model on chromosome 11

Figure 3 presents the results in a Manhattan plot for chromosome 11 ordered by base pair position. The interaction at the midpoint between the corresponding methylation and genetic locus is plotted. The effects tend to be clustered along the chromosome, which is compatible with biological models of gene expression modulation through methylation. This also suggests a further direction of study by exploiting the location of the loci on the chromosome as a further structure to use in the design of the sparse regression, which may, for example, be leveraged by variants such as the fused LASSO [19]. Table 3 reports the top interaction terms. Note that these do not include marginal terms for which the interactions were not selected.

**Table 3:** The most frequently selected interaction terms and whether their component is marginally significant (ie, itself one of the top selected components in the regression)

SNP mark	CpG mark	Selection probability	Marginally significant?
rs4930266	cg00574958	0.20	Yes (CpG)
rs11228481	cg00574958	0.16	No
rs12577789	cg23089549	0.15	Yes (CpG)
rs4930263	cg00574958	0.14	Yes (CpG)
rs748541	cg19261050	0.13	Yes (SNP)
rs17149710	cg00574958	0.12	Yes (CpG)

## Conclusions

We show that sparse regression techniques can be applied to tackle the problems of selecting variables in high-dimensional data sets as often arise in genome-wide or epigenome-wide association studies, including selecting variables in models containing interaction terms. These techniques enable us to improve on the power of multiple F-tests in high dimension as they adopt a more global, instead of locus-by-locus, point of view. The effects seem to appear in a clustered fashion, and further analyses could attempt to leverage the existence of such structure.
